# A Systematic Review and Meta-analysis of the Psychiatric Morbidities and Quality of Life Differences Between Men and Women in Infertile Couples

**DOI:** 10.7759/cureus.37327

**Published:** 2023-04-09

**Authors:** Yaser Mansoor Almutawa, Muneera AlGhareeb, Lateefa Rashed Daraj, Noor Karaidi, Haitham Jahrami

**Affiliations:** 1 Department of Psychiatry, College of Medicine and Medical Sciences, Arabian Gulf University, Manama, BHR; 2 Department of Psychiatry, Psychiatry Hospital, Ministry of Health, Manama, BHR

**Keywords:** stress, quality of life (qol), infertility, depression, anxiety

## Abstract

Infertility is often associated with diverse psychiatric morbidities and quality of life impairments. Hence, this meta-analysis aimed to compare stress, depression, anxiety and quality of life (QoL) among infertile men and women. We retrieved the relevant articles from multiple databases. For the statistical analyses, we used Comprehensive Meta-Analysis software v. 3.7 (Biostat Inc., Englewood, NJ). Standardized mean differences (SMD) with 95% confidence intervals (CI) were calculated and displayed in forest plots. Among the 4123 articles identified, 35 studies met the inclusion criteria. Our results revealed that stress, depression, and anxiety were higher in infertile women compared to men. Similarly, infertile women presented a lower QoL than infertile men. Subgroup analysis revealed that the assessment tool used, study design, and geographical origin were a source of heterogeneity. This meta-analysis showed that psychological disturbances were higher in infertile women compared to men. Physicians need to consider this difference to enable couples to better understand and support each other.

## Introduction and background

The failure to achieve a clinical pregnancy after 12 months of regular treatment or unprotected sexual activity is said to be infertility [1]. Age, lifestyle decisions, and medical issues are just a few variables that might contribute to infertility. Infertility affects 8-12% of reproductive-aged people worldwide [2]. Infertility among young people is the fifth-most serious ailment by the World Health Organization (WHO), and recent data show the problem is worsening. In 2010, the Maternal Health Task Force estimated that 50 million couples were infertile worldwide [3]. Infertility is the failure to conceive despite engaging in regular, unprotected sexual activity for a year (or more) [4]. Infertility is prevalent in South/Central Asia, Sub-Saharan Africa, North Africa/Middle East, and Central/Eastern Europe [5]. Infertility affects men and women equally. Male factors are responsible for one-third of infertility cases in couples, a third are caused by female characteristics, and another one-third are due to male and female reproductive problems or unknown factors [6]. Infertility adversely affects several aspects of a person's life, regardless of which spouse is infertile [7]. There is a strong correlation between infertility and impairments in marital relationships [8], sexual satisfaction [9], and mental well-being [10]. A few detrimental psychological consequences of infertility on infertile couples include stress, depression, and anxiety. These conditions may lengthen the period of infertility and significantly lower the quality of life for couples, ultimately resulting in divorce [11]. According to Monga et al., infertility may aggravate sexual dysfunction and marital strife, lowering the quality of life [12]. Compared to the fertile group, infertile women reported poorer life satisfaction and marital adjustment levels. Due to the constrained scheduling of the encounters around the woman's ovulatory cycle, infertile men demonstrated decreased intercourse satisfaction [12]. The link of sexual intimacy with infertility rather than sexual pleasure has led to many couples reporting diminished enjoyment of sexual intimacy as they undergo treatment [13]. According to several studies, infertile women experience societal stigma, which is a major cause of depression, anxiety, and low self-esteem [14]. Family pressure to procreate also lowers their quality of life [15,16].

Numerous studies have compared psychological morbidity and quality of life among infertile couples. Global reports indicated a remarkable diversity of findings. For example, Peterson et al. revealed a higher rate of anxiety and sexual infertility stress in infertile women than infertile men [17]. However, Wischmann et al. showed that infertile men experience a higher level of anxiety than that experienced by infertile women [18]. On the other hand, some studies revealed no significant difference between men and women [19-21]. Therefore, it is necessary to undertake a meta-analysis to compile the findings of the investigations. The purpose of the present systematic review and meta-analysis was to quantify and compare the overall summary measure of stress, depression, anxiety, and quality of life between infertile men and women.

## Review

Materials and methods

This systematic review and meta-analysis study was carried out according to the Preferred Reporting Items for Systematic Reviews and Meta-Analyses (PRISMA) standards [22] and prospectively registered at PROSPERO, the International prospective register of systematic reviews (CRD42022385084).

Literature Search

To find possibly relevant publications, a thorough investigation was done across several databases, including PubMed/Medline, Scopus, Web of Science, the Cochrane Library, Embase, Google Scholar, PsycINFO, and CINAHL, from the time the databases were created to the end of December 2022. The search strategy was based on the following key search terms: “infertility” OR “sterility” OR “reproductive sterility” OR “subfertility” OR “sub-fertility” AND ‘‘stress’’ OR ‘‘depression’’ OR ‘‘anxiety’’ OR ‘‘quality of life’’ OR ‘‘psychiatric’’ OR ‘‘psychological’’ OR ‘‘psychosocial’’. Two researchers individually carried out each retrieval operation (YMA and MG).

Study Selection

After eliminating duplicates, pertinent articles were scrutinized by title and abstract. Studies that compared any psychological or psychosocial evaluation between infertile men and women were eligible for inclusion. After reviewing the full texts of the remaining studies, eligibility was confirmed.

Inclusion criteria for the studies were as follows: (1) observational studies published in peer-reviewed journals; (2) English-language publications; (3) publications with original findings; (4) evaluation of stress, depression, anxiety, and quality of life as outcomes among infertile men and women; (5) availability of sample size, mean and standard deviation data for both infertile men and women; and (6) validated measures.

Those studies not meeting the following criteria were excluded: (1) those without a full electronic text; (2) those published in a language other than English; (3) those that contain only a limited amount of outcome information; and (4) letters, editorials, comments, protocols, review papers (including systematic reviews and meta-analyses), and guidelines.

Data Extraction

Following the inclusion and exclusion criteria, two independent reviewers (YMA and MG) gathered data from the relevant papers. We recorded the data on a standardized data sheet, including study and year of publication, study design, country, sample size, age of participants, duration of infertility, outcomes, and measures. Data were checked for accuracy by the third and fourth authors (LRD and NK), who also served as referees in case of disagreements.

Measures

This meta-analysis was composed of 12 measures that later were divided into four groups: stress, depression, anxiety, and quality of life.

Infertility-Related Stress

The Fertility Problem Inventory (FPI) was used to measure the stress associated with infertility. FPI was created by Newton et al. to evaluate the degree of disruption and stress that the fertility issue had caused generally and in connection with three domains (personal, social, and marital) [23].

Depression Outcomes

Depression outcomes were assessed by four measures:

(i) the Hospital Anxiety and Depression Scale (HADS-D); (ii) the 21-item Hamilton Rating Scale for Depression (HAM-D), a scale used to rate depression - the following are the threshold scores: >25 severe, 18-24 moderate, 8-17 mild, <7 no depression [24]; (iii) the Symptom Checklist-90-Revised (SCL-90-R); (iv) the Beck Depression Inventory (BDI) [25], which contains 21 questions, with scores ranging from 0 to 3. Scores under 10 indicate a lack of depression; scores between 11 and 18 indicate mild depression; scores between 19 and 29 indicate moderate depression; and scores over 30 indicate severe depression.

Anxiety Outcomes

Anxiety outcomes were assessed by four measures:

Hospital Anxiety and Depression Scale (HADS-A): This scale was developed by Zigmond and Snaith in 1983. There are seven scales connected to anxiety (HADS-A) and seven scales related to depression (HADS-D). The HADS is regarded as a potent, reliable tool for assessing anxiety and depression. High scores reflect higher levels of anxiety and despair [26].

Hamilton Rating Scale for Anxiety (HAM-A): It consists of 14 items, each of which receives a score between 0 (absence) and 4 (very severe). The following are the cutoff scores: 0-5 no anxiety, 6-14 mild anxiety, and 15 or more severe anxiety [27].

Symptom Checklist-90-Revised (SCL-90-R): This is a self-report symptom assessment to assess psychological symptoms and distress. It has 90 items with responses ranging from zero (not at all) to four (extremely) on a five-point scale [28];

Beck Anxiety Inventory (BAI): Describes various anxiety symptoms. The total point range of 0 to 63 allows for the easy classification of anxiety into three categories: extremely low (normal) (0 to 21 points), moderate (22 to 35 points), and severe (above 35 points), which indicates the need for specialized medical consultation [29].

Quality of Life

Quality of life was assessed by three measures:

FertiQoL: This measure has two modules, the core and therapy parts, and 36 elements. A higher score on any subscale of the six produced by the FertiQoL indicates a better quality of life [30].

World Health Organization Quality of Life-BREF: This is a general QOL evaluation tool made up of four areas, physical health, psychological health, social interactions, and the environment [31].

Short Form Health Survey (SF-36): This is a 36-item, patient-reported survey of patient health. An overall score is a number between 0 and 100. A higher score denotes a higher living quality [32].

Quality assessment of the studies

The Newcastle-Ottawa Scale (NOS), which assesses selection bias, comparability of exposed and control participants, and outcome evaluation, was used to evaluate the quality of non-randomized research. Each criterion was given a star rating of 1 or 0 stars. For case-control and observational studies (prospective or retrospective), the NOS checklist's overall star rating ranged from 0 to 9 stars, while for cross-sectional studies, it went from 0 to 10 stars.

The NOS instrument assesses three areas: (1) study group selection (maximum of four stars for case-control and observational studies (prospective or retrospective) and five stars for cross-sectional studies), (2) study group comparability (maximum of two stars), and (3) outcome assessment (max three stars). Two authors evaluated quality independently, and disagreements were settled by discussion. A study with a score of 7 to 9 or 10 has good quality, a score of 4 to 6 is fair quality, and a score of 0 to 3 is poor quality [33].

Statistical analysis

We used the Comprehensive Meta-Analysis software v. 3.7 (Biostat Inc., Englewood, NJ) for statistical evaluations. We generated the standardized mean difference (SMD) with 95% confidence intervals (CIs) to assess all the results. Later we interpreted the SMD values as follows:

(i) SMD <0: women experienced more psychiatric comorbidities than men (i.e., the male group had a lower mean score than the women group); (ii) SMD = 0: No difference between men and women; (iii) SMD >0: men experienced more psychiatric comorbidities than women (i.e., the male group had a higher mean score than the women group).

A p-value of 0.05 was set as the level of significance. We used the Cochrane chi-squared test to assess article heterogeneity; a p-value of 0.05 or higher indicates the presence of heterogeneity. Using the I^2 ^value, we determined the impact of heterogeneity on the meta-analysis. The pooled studies exhibited moderate to high levels of heterogeneity, as indicated by I^2^ values >50% and p <0.05. We used the fixed-effects design if I^2^ <50% and p >0.05; otherwise, a random-effects method was utilized [34]. Furthermore, we performed subgroup analyses and sensitivity analyses to identify sources of heterogeneity. To assess publication bias, we used Egger's test. This latter was further considered by looking at the funnel plots' symmetry.

Results

Identification of Studies

**Figure 1:**
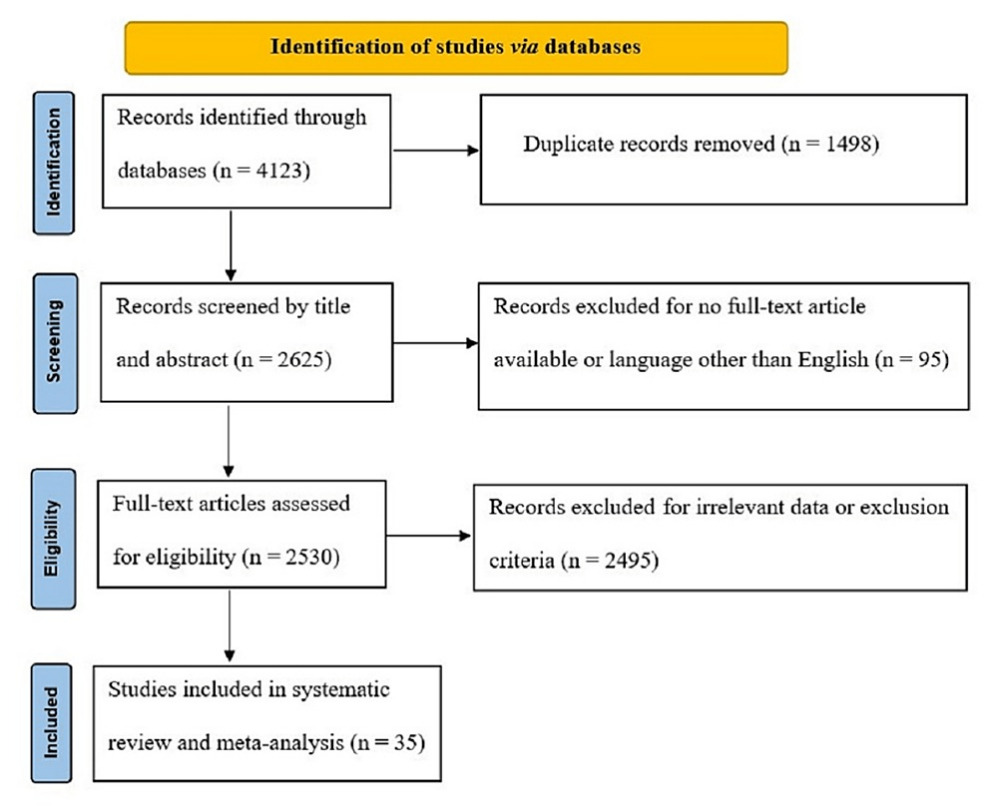
PRISMA flow diagram

We identified 4123 studies in the databases that needed screening; later, 2625 abstracts were possibly eligible for full-text analysis. Thirty-five studies included in this systematic review and the meta-analysis study satisfied the eligibility requirements. Figure 1 displays the PRISMA flow diagram.

Characteristics of Included Studies

The included studies were from 18 countries and were published between 2001 and 2022. The 35 publications that comprised this systematic review and meta-analysis included 29 cross-sectional studies, four prospective cohort studies, and two case-control studies. The number of couples in the sample ranged from 26 to 818 in the included publications. The mean age of men varied from 29.00 ± 3.50 years to 41.60 ± 5.90 years, while the mean age of women varied from 27.48 ± 4.21 years to 40.80 ± 4.70 years. The duration of infertility ranged from 1.73 ± 1.15 years to 7.44 ± 5.30 years. Infertility-related stress was evaluated in 11 studies, and 20 and 14 studies investigated depression and anxiety outcomes, respectively. However, 14 studies assessed the quality of life. Overall, the scores of the included studies ranged from five to nine stars. Among the included studies, 31 scored good quality, and four articles scored fair quality. Table 1 summarizes the characteristics of the studies.

**Table 1:** Characteristics of the included studies BDI: Beck Depression Inventory; BAI: Beck Anxiety Inventory; FPI: Fertility Problem Inventory; SCL-90-R: Symptom Checklist-90-Revised; HAM-D: Hamilton Rating Scale for Depression; HAM-A: HAM-Anxiety; HADS: Hospital Anxiety and Depression Scale; QOL: Quality of life; WHO: World Health Organization; SN: Study number; NOS: Newcastle-Ottawa scale; ND: not defined ** Two groups; *** Three groups

SN	Study and year of publication	Study design	Country	Sample size		Age of participant, years Mean ± SD		Duration of infertility, years Mean ± SD	Outcomes and measures	NOS
				Men	Women	Men	Women			
1	Mahadeen et al., 2018 [7]	Cross-sectional	Jordan	103	145	ND	ND	ND	Depression (BDI)	7(Good)
2	Peterson et al., 2007 [17]	Prospective cohort	Canada	295	306	34.50 ± 5.70	32.40 ± 4.20	ND	Anxiety (BAI); Stress (FPI)	6(Fair)
3	Wischmann et al., 2001 [18]	Cross-sectional	Germany	512	536	34.30	32.10	4.20 ± 2.30; 4.30 ± 2.70 **	Depression (SCL-90-R); Anxiety (SCL-90-R)	7(Good)
4	Cserepes et al., 2013 [19]	Cross-sectional	Hungary	26	27	33.50 ± 4.65	29.89 ± 4.05	2.65 ± 1.48 (women) 2.86 ± 1.62 (men)	Stress (FPI); Depression (BDI)	7(Good)
5	Madero et al., 2017 [20]	Cross-sectional	Spain	201	347	41.60 ± 5.90	40.80 ± 4.70	ND	Quality of life (FertiQoL); Depression (HAD-D); Anxiety (HAD-A)	7(Good)
6	Fernandes et al., 2021 [21]	Cross-sectional	Portugal	63	63	35.17 ± 4.33	35.17 ± 4.33	3.27 ± 2.63	Depression (HAD-D); Anxiety (HAD-A)	9(Good)
7	Boivin and Schmidt, 2005 [35]	Prospective cohort	Denmark	818	818	33.80± 5.10	31.50 ± 3.50	4.09 ± 2.12	Stress (FPI)	6(Fair)
8	Bose et al., 2021 [36]	Cross‑sectional	India	100	100	31.20 ± 4.41	27.48 ± 4.21	2.51 ± .0.63	Quality of life (FertiQoL); Stress (FPI)	7(Good)
9	Chachamovich et al., 2009 [37]	Cross‑sectional	Brazil	162	162	36.15 ± 7.69	32.11 ± 5.80	5.76 ± 3.64	Quality of life (WHOQoL-BREF); Depression (BDI)	7(Good)
10	Chachamovich et al., 2010 [38]	Cross-sectional	Brazil	162	162	36.15 ± 7.69	32.11 ± 5.80	5.76 ± 3.64	Quality of life (WHOQoL-BREF); Depression (BDI)	7(Good)
11	Dadkhahtehrani et al., 2018 [39]	Cross-sectional	Iran	200	200	32.61 ± 5.32	28.82 ± 5.13	4.93 ± 3.95	Quality of life (SF‑36)	8(Good)
12	Donarelli et al., 2015 [40]	Cross-sectional	Italy	459	459	37.06 ± 5.22	34.18 ± 4.69	ND	Stress (FPI)	8(Good)
13	Donarelli et al., 2016 [41]	Cross-sectional	Italy	288	301	37.80 ± 5.70	34.90 ± 5.03	4.00 ± 3.37	Quality of life (FertiQoL)	8(Good)
14	Drosdzol and Skrzypulec, 2009 [42]	Cross-sectional	Poland	188	206	31.40 ± 4.70	29.80 ± 4.10	3.04 ± 3.34	Depression (BDI); Anxiety (BAI)	8(Good)
15	El Kissi et al., 2013 [43]	Cross-sectional	Tunisia	100	100	38.74 ± 5.87	32.69 ± 4.91	5.19 ± 4.62	Depression (HAD-D); Anxiety (HAD-A)	8(Good)
16	El Kissi et al., 2014 [44]	Case-control	Tunisia	100	100	38.74 ± 5.87	32.69 ± 4.91	5.19 ± 4.62	Quality of life (SF‑36)	7(Good)
17	Fassino et al., 2002 [45]	Case-control	Italy	85	85	33.69 ± 4.63; 31.51 ± 4.46**	30.89 ± 4.27; 29.37 ± 3.70**	ND	Depression (HAM-D); Anxiety (HAM-A)	7(Good)
18	Goker et al., 2017 [46]	Cross-sectional	Turkey	127	127	31.40 ± 5.90	27.50 ± 5.40	3.80 ± 3.30	Quality of life (FertiQoL)	8(Good)
19	Herrmann et al., 2011 [47]	Cross-sectional	Germany	199	199	35.60	33.00	4.50	Quality of life (WHOQoL-BREF)	7(Good)
20	Karimzadeh et al., 2017 [48]	Cross-sectional	Iran	78	50	31.68 ± 3.43	28.30 ± 5.96	5.90 ± 4.19 (women) 5.50 ± 2.76 (men)	Depression (SCL-90-R); Anxiety (SCL-90-R)	8(Good)
21	Kim et al., 2016 [49]	Cross-sectional	South Korea	121	121	ND	ND	ND	Quality of life (FertiQoL); Stress (FPI); Depression (BDI)	9(Good)
22	Lei et al., 2021 [50]	Cross-sectional	China	508	508	31.06 ± 4.18	29.32 ± 3.90	3.10 ± 2.73 (women) 3.45 ± 2.08 (men)	Stress (FPI)	9(Good)
23	Maroufizadeh et al., 2015 [51]	Cross-sectional	Iran	122	208	33.90 ± 5.30	30.30 ± 5.40	6.20 ± 4.10	Depression (HAD-D); Anxiety (HAD-A)	7(Good)
24	Maroufizadeh et al., 2018 [52]	Cross-sectional	Iran	141	141	34.92 ± 6.35	29.82 ± 6.00	4.85 ± 3.76	Depression (HAD-D)	7(Good)
25	Navid et al., 2017 [53]	Cross-sectional	Iran	248	248	33.25 ± 5.70	29.15 ± 5.28	4.82 ± 3.50	Depression (HAD-D); Anxiety (HAD-A)	7(Good)
26	Ngai and Loke, 2021 [54]	Cross-sectional	Hong Kong	135	135	36.00 ± 5.50	33.80 ± 3.60	1.73 ± 1.15	Quality of life (FertiQoL); Stress (FPI)	7(Good)
27	Patel et al., 2018 [55]	Cross-sectional	India	81	81	29.00 ± 3.50	35.00 ± 4.30	4.00 ± 2.50	Depression (HAM-D); Anxiety (HAM-A); Stress (FPI)	8(Good)
28	Pedro et al., 2017 [56]	Prospective cohort	Portugal	139	139	33.56 ± 5.61	31.76 ± 4.73	2.32 ± 2.07	Depression (BDI); Stress (FPI)	6(Fair)
29	Peterson et al., 2003 [57]	Prospective cohort	Canada	525	525	33.80	32.30	3.50	Depression (BDI); Stress (FPI)	6(Fair)
30	Van Rooij et al., 2007 [58]	Cross-sectional	Netherlands	142	161	33.46 ± 7.78; 35.60 ± 5.16; 37.92 ± 5.38 ***	29.26 ± 6.73; 33.00 ± 5.09; 34.89 ± 4.03 ***	4.21 ± 4.55 (women) 6.50 ± 4.81 (women) 6.27 ± 3.75 (women) 3.10 ± 3.26 (men) 7.44 ± 5.30 (men) 6.14 ± 4.47 (men)	Depression (SCL-90-R); Anxiety (SCL-90-R)	7(Good)
31	Wadadekar et al., 2021 [59]	Cross-sectional	India	137	137	ND	ND	ND	Quality of life (FertiQoL)	7(Good)
32	Wang et al., 2022 [60]	Cross-sectional	China	428	428	32.42 ± 5.19	31.00 ± 1.24	4.44 ± 3.21	Quality of life (FertiQoL)	8(Good)
33	Wischmann et al., 2009 [61]	Cross-sectional	Germany	535	633	34.87 ± 5.45; 35.31 ± 5.22 **	32.43 ± 4.26; 33.45 ± 3.98 **	4.36 ± 3.01 (women) 4.50 ± 2.70 (women) 4.32 ± 3.07 (men) 4.34 ± 2.62 (men)	Depression (SCL-90-R); Anxiety (SCL-90-R	7(Good)
34	Yoldemir et al., 2021 [62]	Cross-sectional	Turkey	320	320	32.36 ± 6.06; 32.67 ± 6.35 **	27.69 ± 4.02; 29.48 ± 3.38 **	4.31 ± 2.45; 3.82 ± 2.60 **	Anxiety (HAM-A)	7(Good)
35	Zurlo et al., 2018 [63]	Cross-sectional	Italy	206	206	34.00 ± 3.85	34.00 ± 3.85	3.00 ± 2.40	Quality of life (FertiQoL)	8(Good)

Outcomes

**Figure 2:**
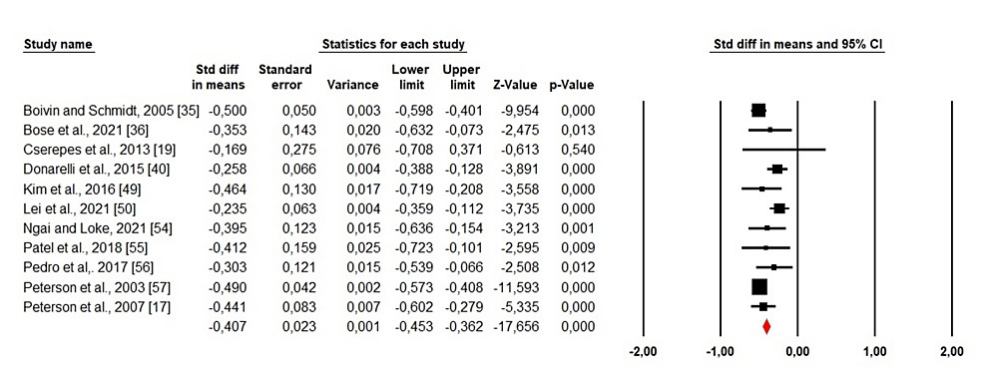
Forest plot showing the estimated standardized mean difference (SMD) of stress between men and women Boivin and Schmidt, 2005 [35]; Bose et al., 2021 [36]; Cserepes et al., 2013 [19]; Donarelli et al., 2015 [40]; Kim et al., 2016 [49]; Lei et al., 2021 [50]; Ngai and Loke, 2021 [54]; Patel et al., 2018 [55]; Pedro et al., 2017 [56]; Peterson et al., 2003 [57]; Peterson et al., 2007 [17].

Stress: 11 studies evaluated stress among men and women. We used a random-effects design due to the high heterogeneity (Chi^2^ = 21,82, p = 0.016, I^2^ = 54,18%). The forest plot found that SMD was <0. which indicated that women experienced much more stress than men (SMD: -0.407; 95% CI: -0.453 - -0.362; p <0.001) (Figure 2).

**Figure 3:**
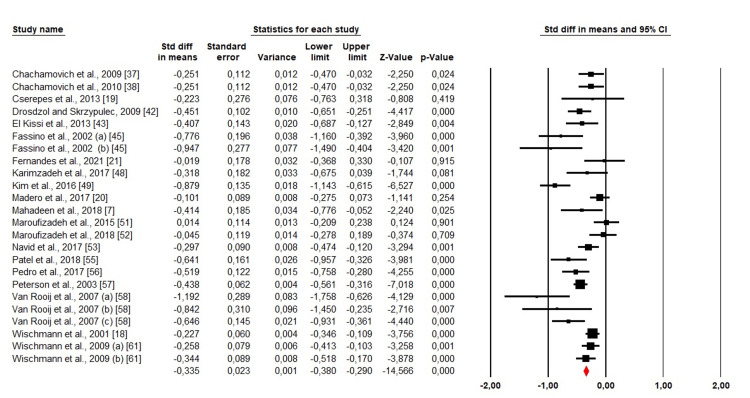
Forest plot showing the estimated standardized mean difference (SMD) of depression between men and women Chachamovich et al., 2009 [37]; Chachamovich et al., 2010 [38]; Cserepes et al., 2013 [19]; Drosdzol and Skrzypulec, 2009 [42]; El Kissi et al., 2013 [43]; Fassino et al., 2002 (a) organic infertility, (b) functional infertility [45]; Fernandes et al., 2021 [21]; Karimzadeh et al., 2017 [48]; Kim et al., 2016 [49]; Madero et al., 2017 [20]; Mahadeen et al., 2018 [7]; Maroufizadeh et al., 2015 [51]; Maroufizadeh et al., 2018 [52]; Navid et al., 2017 [53]; Patel et al., 2018 [55]; Pedro et al., 2017 [56]; Peterson et al., 2003 [57]; Van Rooij et al., 2007 (a) Turkish migrants, (b) Turkish people living in western Turkey, (c) Dutch [58]; Wischmann et al., 2001 [18]; Wischmann et al., 2009 (a): Not counseled and (b): Taking up counseling [61].

Depression: Among the 35 included studies, 20 studies, including 24 cohorts, have evaluated depression among men and women. We used a random-effects design due to the high heterogeneity (Chi^2^ = 83,70. p <0.001, I^2^ = 72,52%). The forest plot revealed that SMD was <0, indicating that women were much more likely than men to experience depression (SMD: -0.335; 95% CI: -0.380 - -0.290; p <0.001) (Figure 3).

**Figure 4:**
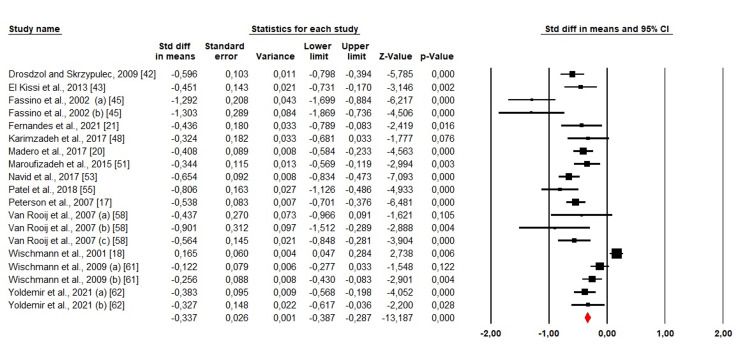
Forest plot showing the estimated standardized mean difference (SMD) of anxiety between men and women Drosdzol and Skrzypulec, 2009 [42]; El Kissi et al., 2013 [43]; Fassino et al., 2002 (a) organic infertility, (b) functional infertility [45], Fernandes et al., 2021 [21]; Karimzadeh et al., 2017 [48]; Madero et al., 2017 [20]; Maroufizadeh et al., 2015 [51]; Navid et al., 2017 [53]; Patel et al., 2018 [55]; Peterson et al., 2007 [17]; Van Rooij et al., 2007 (a) Turkish migrants, (b) Turkish people living in western Turkey, (c) Dutch [58]; Wischmann et al., 2001 [18]; Wischmann et al., 2009 (a) Not counseled, (b) taking up counseling [61], Yoldemir et al., 2021 (a) primary infertile; (b) secondary infertile [62].

Anxiety: Among the 35 included studies, 14 (19 cohorts) evaluated anxiety among men and women. We used a random-effects design because the heterogeneity was high (Chi^2 ^= 149,64, p <0.001, I^2^ = 87.97%). The analysis revealed that SMD was <0. which indicated that anxiety was far higher in women than in men (SMD: -0.337; 95% CI: -0.387 - -0.287; p <0.001) (Figure 4).

**Figure 5:**
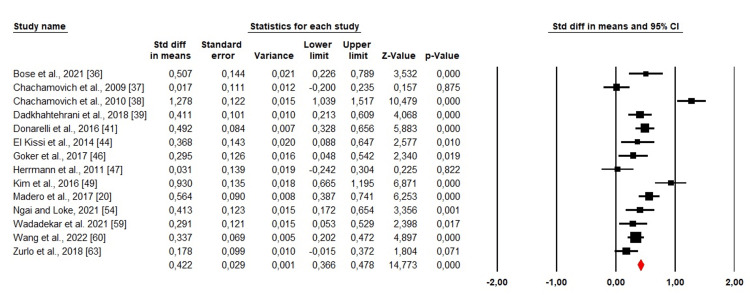
Forest plot showing the estimated standardized mean difference (SMD) of quality of life between men and women Bose et al., 2021 [36]; Chachamovich et al., 2009 [37]; Chachamovich et al., 2010 [38]; Dadkhahtehrani et al., 2018 [39]; Donarelli et al., 2016 [41]; El Kissi et al., 2014 [44]; Goker et al., 2017 [46]; Herrmann et al., 2011 [47]; Kim et al., 2016 [49]; Madero et al., 2017 [20]; Ngai and Loke, 2021 [54]; Wadadekar et al., 2021 [59]; Wang et al., 2022 [60]; Zurlo et al., 2018 [63].

Quality of life: Among the 35 included studies, 14 were evaluated for the quality of life among men and women. We used a random-effects design due to the high heterogeneity (Chi^2^ = 97,95, p = 0. 000. I^2^ = 86.72%). The forest plot demonstrated that SMD was >0. which indicated that men's quality of life was much higher than women's (SMD: 0.422; 95% CI: 0.366 - 0.478; p <0.001) (Figure 5).

**Figure 6:**
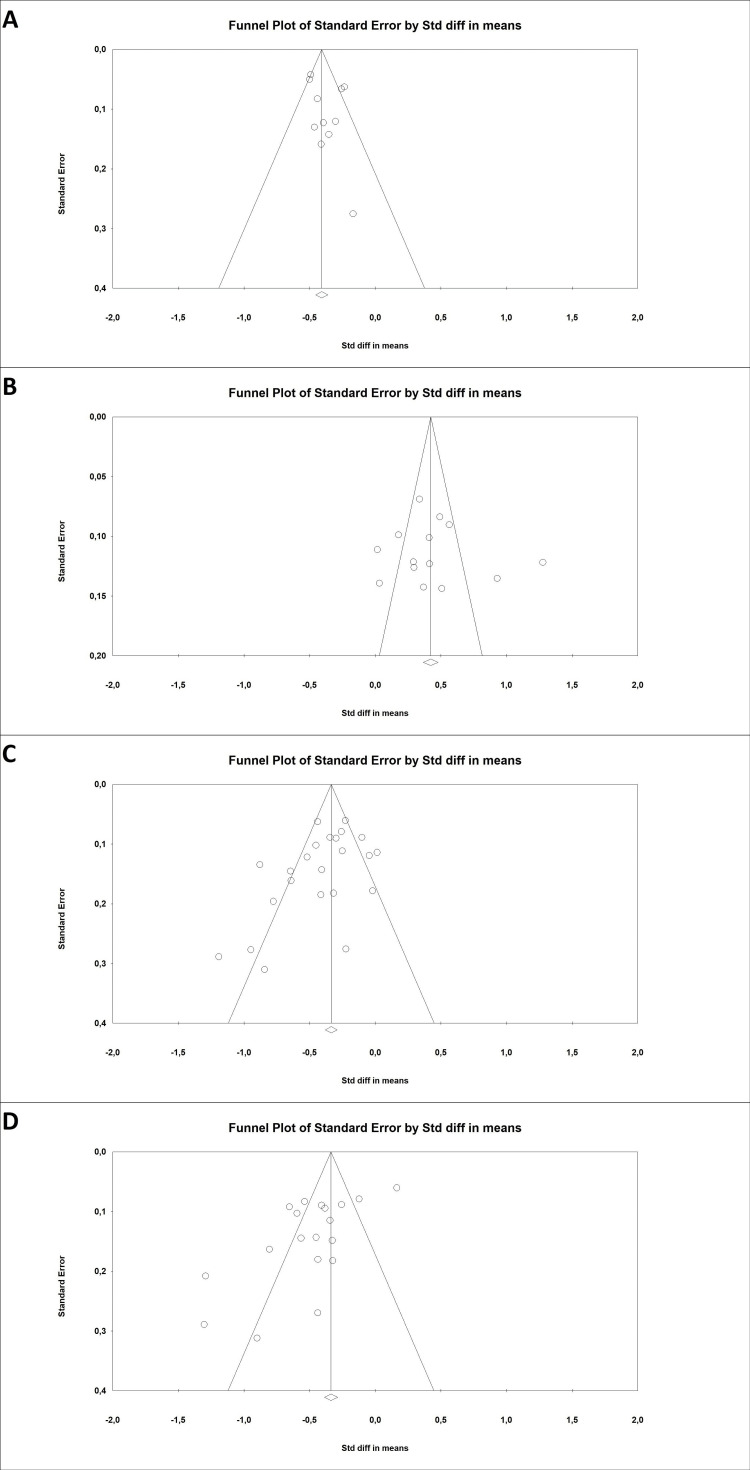
The included articles' funnel plots show no evidence of publication bias in terms of (A) stress and (B) quality of life and evidence of publication bias in terms of (C) depression and (D) anxiety scores

Publication bias: Egger’s test was not statistically significant for stress and quality of life (p = 0.139, p = 0.342, respectively), indicating the absence of publication bias. This finding was confirmed by the funnel plot (Figures 6A-6B). However, Egger’s test was statistically significant for depression and anxiety (p = 0.046, p = 0.003), indicating publication bias. The funnel plot confirmed this finding (Figures 6C-6D). 

Subgroup Analysis

The geographical origin of the work, the assessment technique used to assess outcomes, and the study's design all impacted the standardized mean difference of anxiety, depression, quality of life, and stress between infertile men and women.

Stress: The work's geographical origin and the study design were sources of heterogeneity for the stress outcome (p = 0.020, p <0.001, respectively). Indeed, Asia had the highest standardized mean stress difference between infertile men and women (SMD = -0.314), while North America showed the lowest standardized mean difference (SMD = -0.480). Similarly, the standardized mean difference of stress was significantly higher in cross-sectional studies (SMD = -0.293) than in prospective cohort studies (SMD = -0.476) (Table 2).

**Table 2:** Subgroup analyses for (A) stress, (B) depression, (C) anxiety, and (D) quality of life outcomes HADS: Hospital Anxiety and Depression Scale; BDI: Beck Depression Inventory; HAM-D: Hamilton Rating Scale for Depression; SCL-90-R: Symptom Checklist-90-Revised; BAI: Beck Anxiety Inventory; HAM-A: HAM-Anxiety; QOL: Quality of life; WHO: World Health Organization

Subgroups	No. of cohorts	Standardized mean difference	95% confidence interval	Heterogeneity		
				I^2^	Chi^2^	p
A-Stress						
Geographic origin						
Europe	4	-0.396	-0.470 — -0.322	69,68%	9,896	0.020
Asia	5	-0.314	-0.404 — -0.223	0%	3,774	
North America	2	-0.480	-0.553 — -0.406	0%	0.289	
Study design						
Cross-sectional	7	-0.293	-0.367 — -0.219	0%	4,466	<0.001
Prospective cohort	4	-0.476	-0.533 — -0.419	0%	2,593	
B-Depression						
Geographic origin						
Europe	13	-0.326	-0.388 — -0.264	72,65%	43,89	0.358
Asia	7	-0.315	-0.410 — -0.220	83,07%	35,44	
Africa	1	-0.407	-0.687 — -0.126	0%	0.00	
North America	1	-0.438	-0.560 — -0.315	0%	0.00	
South America	2	-0.251	-0.406 — -0.096	0%	0.00	
Assessment tool used						
HADS-D	6	-0.150	-0.240 — -6,092	47,90%	17,41	<0.001
BDI	8	-0.434	-0.509 — -0.359	59,80%	9,59	
HAM-D	3	-0.737	-0.960 — -0.515	0%	0.96	
SCL-90-R	7	-0.318	-0.394 — -0.242	70.02%	20.01	
Study design						
Cross-sectional	20	-0.296	-0.346 — -0.246	71,42%	66,48	<0.001
Case-control	2	-0.833	-1,146 — -0.519	0%	0.25	
Prospective cohort	2	-0.455	-0.564 — -0.346	0%	0.34	
C-Anxiety						
Geographic origin						
Europe	13	-0.253	-0.313 — -0.193	89,61%	115,59	<0.001
Asia	4	-0.547	-0.668 — -0.426	64,64%	8,48	
Africa	1	-0.450	-0.731 — -0.170	0%	0	
North America	1	-0.538	-0.701 — -0.375	0%	0	
Assessment tool used						
HAD-A	5	-0.475	-0.574 — -0.377	29,79%	5,69	<0.001
BAI	2	-0.561	-0.687 — -0.434	0%	0.19	
HAM-A	5	-0.580	-0.709 — -0.450	85,27%	27,15	
SCL-90-R	7	-0.895	-0.164 — -1,292	85,85%	42,41	
Study design						
Cross-sectional	16	-0.290	-0.343 — -0.237	86,18%	108,56	<0.001
Case-control	2	-1,295	-1,626 — -0.964	0%	9,83	
Prospective cohort	1	-0.538	-0.701 — -0.375	0%	0.00	
D-Quality of life						
Geographic origin						
Europe	5	0.368	0.279-0.458	76,20%	16,80	0.129
Asia	6	0.428	1,832-0.344	70.78%	17,11	
Africa	1	0.367	8,805-0.647	0%	0.00	
South America	2	0.589	0.428- 0.750	98,28%	58,35	
Assessment tool used						
WHOQOL-Bref	3	0.445	0.306-0.584	97,15%	70.25	0.902
FertiQoL	9	0.420	0.354-0.486	70.83%	27,43	
SF-36	2	0.396	0.234-0.558	0%	6,19	
Study design						
Cross-sectional	13	0.424	0.367-0.481	87,73%	97,80	0.697
Case-control	1	0.367	8,805-0.647	0%	0.00	

Depression: Regarding depression, there was no significant difference between the different continents (p = 0.358). However, the tool used for assessment and the study design was a source of heterogeneity (p <0.001). Indeed, the HADS-D scale revealed significantly higher SMD between infertile men and women (SMD = -0.150) than the HAM-D scale (SMD = -0.737). Moreover, the SMD of depression was significantly higher in cross-sectional studies (SMD = -0.296) than in case-control studies (SMD = -0.833) (Table 2).

Anxiety: When we used the geographical origin of the work as a moderator, the SMD of anxiety significantly differed between studies (p <0.001). Indeed, the highest standardized mean difference in anxiety between infertile men and women was detected in Europe (SMD = -0.253), followed by Africa (SMD = -0.450). Moreover, the SMD of anxiety significantly differed according to the tool used for assessment (p <0.001). The SMD of anxiety was very high (SMD = -0.475) when measured using HADS-A, compared with SCL-90 (SMD = -0.895). The SMD of anxiety significantly differed depending on the study design. Indeed, the SMD of anxiety was higher in cross-sectional studies (SMD = -0.290) than in prospective cohort (SMD = -0.538) and case-control (SMD = -1,295) studies (Table 2).

Quality of life: The SMD of quality of life did not significantly differ between studies when we set the geographical origin of the work as a moderator (p = 0.129). Indeed, the highest SMD of quality of life between infertile men and women was detected in South America (SMD = 0.589), while the lowest SMD was revealed in Africa (SMD = 0.367). However, the assessment tool and the study design did not constitute a source of heterogeneity (p = 0.902 and p = 0.697, respectively) (Table 2).

Sensitivity Analysis

To further pinpoint the potential source of heterogeneity in the pooled analysis of the SMD of stress, depression, anxiety, and quality of life outcomes between infertile men and women, a sensitivity analysis was carried out. The results showed no significant differences, proving the reliability of the meta-analysis. The SMD of stress, depression, anxiety, and quality of life outcomes ranged from -0.434 (95% CI -0.482 - -0.385) to -0.372 (95% CI -0.426 - -0.318), from -0.351 (95% CI -0.398 - -0.304) to -0.318 (95% CI -0.364 - -0.272), from -0.446 (95% CI -0.501 - -0.391) to -0.310 (95% CI -0.362 - -0.258), and from 0.372 (95% CI 0.314-0.429) to 0.450 (95% CI 0.392 - 0.508), respectively, in the leave-one-out sensitivity analysis (Table 3).

**Table 3:** Sensitivity analysis for (A) stress, (B) depression, (C) anxiety, and (D) quality of life outcomes

Study removed	SMD (95% CI)	p
A-Stress		
Peterson et al., 2007 [17]	-0.404 (-0.451 — -0.357)	<0.001
Cserepes et al., 2013 [19]	-0.409 (-0.454 — -0.363)	<0.001
Boivin and Schmidt, 2005 [35]	-0.382 (-0.433 — -0.331)	<0.001
Bose et al., 2021 [36]	-0.408 (-0.454 — -0.363)	<0.001
Donarelli et al., 2015 [40]	-0.428 (-0.476 — -0.379)	<0.001
Kim et al., 2016 [49]	-0.405 (-0.451 — -0.359)	<0.001
Lei et al., 2021 [50]	-0.434 (-0.482 — -0.385)	<0.001
Ngai and Loke, 2021 [54]	-0.407 (-0.453 — -0.361)	<0.001
Patel et al., 2018 [55]	-0.407 (-0.453 — -0.361)	<0.001
Pedro et al., 2017 [56]	-0.411 (-0.457 — -0.365)	<0.001
Peterson et al., 2003 [57]	-0.372 (-0.426 — -0.318)	<0.001
B-Depression		
Mahadeen et al., 2018 [7]	-0.333 (-0.378 — -0.288)	<0.001
Wischmann et al., 2001 [18]	-0.352 (-0.401 — -0.304)	<0.001
Cserepes et al., 2013 [19]	-0.335 (-0.380 — -0.290)	<0.001
Madero et al., 2017 [20]	-0.351 (-0.398 — -0.304)	<0.001
Fernandes et al., 2021 [21]	-0.340 (-0.385 — -0.294)	<0.001
Chachamovich et al., 2009 [37]	-0.338 (-0.384 — -0.292)	<0.001
Chachamovich et al., 2010 [38]	-0.338 (-0.384 — -0.292)	<0.001
Drosdzol and Skrzypulec, 2009 [42]	-0.328 (-0.374 — -0.282)	<0.001
El Kissi et al., 2013 [43]	-0.332 (-0.378 — -0.287)	<0.001
Fassino et al., 2002 (a) [45]	-0.328 (-0.374 — -0.283)	<0.001
Fassino et al., 2002 (b) [45]	-0.330 (-0.375 — -0.285)	<0.001
Karimzadeh et al., 2017 [48]	-0.335 (-0.380 — -0.289)	<0.001
Kim et al., 2016 [49]	-0.318 (-0.364 — -0.272)	<0.001
Maroufizadeh et al., 2015 [51]	-0.349 (-0.395 — -0.303)	<0.001
Maroufizadeh et al., 2018 [52]	-0.346 (-0.391 — -0.300)	<0.001
Navid et al., 2017 [53]	-0.337 (-0.383 — -0.290)	<0.001
Patel et al., 2018 [55]	-0.328 (-0.373 — -0.282)	<0.001
Pedro et al., 2017 [56]	-0.328 (-0.373 — -0.282)	<0.001
Peterson et al., 2003 [57]	-0.318 (-0.367 — -0.270)	<0.001
Van Rooij et al., 2007 (a) [58]	-0.329 (-0.374 — -0.284)	<0.001
Van Rooij et al., 2007 (b) [58]	-0.331 (-0.377 — -0.286)	<0.001
Van Rooij et al., 2007 (c) [58]	-0.326 (-0.372 — -0.281)	<0.001
Wischmann et al., 2009 (a) [61]	-0.341 (-0.388 — -0.294)	<0.001
Wischmann et al., 2009 (b) [61]	-0.334 (-0.380 — -0.287)	<0.001
C-Anxiety		
Peterson et al., 2007 [17]	-0.315 (-0.368 — -0.263)	<0.001
Wischmann et al., 2001 [18]	-0.446 (-0.501 — -0.391)	<0.001
Madero et al., 2017 [20]	-0.330 (-0.382 — -0.278)	<0.001
Drosdzol and Skrzypulec, 2009 [42]	-0.319 (-0.371 — -0.268)	<0.001
El Kissi et al., 2013 [43]	-0.333 (-0.384 — -0.282)	<0.001
Fassino et al., 2002 (a) [45]	-0.322 (-0.372 — -0.271)	<0.001
Fassino et al., 2002 (b) [45]	-0.329 (-0.379 — -0.279)	<0.001
Fernandes et al., 2021 [48]	-0.334 (-0.385 — -0.284)	<0.001
Karimzadeh et al., 2017 [48]	-0.337 (-0.387 — -0.286)	<0.001
Maroufizadeh et al., 2015 [51]	-0.336 (-0.387 — -0.285)	<0.001
Navid et al., 2017 [53]	-0.310 (-0.362 — -0.258)	<0.001
Patel et al., 2018 [55]	-0.325 (-0.375 — -0.274)	<0.001
Van Rooij et al., 2007 (a) [58]	-0.336 (-0.386 — -0.285)	<0.001
Van Rooij et al., 2007 (b) [58]	-0.333 (-0.383 — -0.282)	<0.001
Van Rooij et al., 2007 (c) [58]	-0.329 (-0.380 — -0.278)	<0.001
Wischmann et al., 2009 (a) [61]	-0.362 (-0.415 — -0.309)	<0.001
Wischmann et al., 2009 (b) [61]	-0.344 (-0.396 — -0.291)	<0.001
Yoldemir et al., 2021 (a) [62]	-0.333 (-0.385 — -0.281)	<0.001
Yoldemir et al., 2021 (b) [62]	-0.337 (-0.388 — -0.286)	<0.001
D-Quality of life		
Madero et al., 2017 [20]	0.406 (0.347-0.465)	<0.001
Bose et al., 2021 [36]	0.418 (0.361-0.475)	<0.001
Chachamovich et al., 2009 [37]	0.450 (0.392-0.508)	<0.001
Chachamovich et al., 2010 [38]	0.372 (0.314-0.429)	<0.001
Dadkhahtehrani et al., 2018 [39]	0.422 (0.364-0.481)	<0.001
Donarelli et al., 2016 [41]	0.412 (0.353-0.472)	<0.001
El Kissi et al., 2014 [44]	0.424 (0.367-0.481)	<0.001
Goker et al., 2017 [46]	0.428 (0.371-0.486)	<0.001
Herrmann et al., 2011 [47]	0.439 (0.381-0.496)	<0.001
Kim et al., 2016 [49]	0.398 (0.340-0.455)	<0.001
Ngai and Loke, 2021 [54]	0.422 (0.364-0.480)	<0.001
Wadadekar et al., 2021 [59]	0.429 (0.372-0.487)	<0.001
Wang et al., 2022 [60]	0.439 (0.378-0.501)	<0.001
Zurlo et al., 2018 [63]	0.444 (0.385-0.502)	<0.001

Discussion

Infertility is a global health concern linked to emotional, mental, and social issues. This study is the first systematic review and meta-analysis that compares the stress, quality of life, anxiety, and depression levels of infertile men and women, as far as we are aware. Notably, all the papers under consideration are from 2001 or later, indicating that the field of research on the stress, anxiety, and depression of infertile people is relatively new. This investigation produced significant findings. Research repeatedly demonstrates that women experience anxiety and depression at higher rates than men (p<0.05). Numerous studies have confirmed this disparity [21,42,45,48,49]. This fact is not surprising as infertile women are more frequently influenced by their husbands, families, and society because their inability to conceive has a greater negative psychological effect on their conduct than infertile men [64].

Consequently, merging therapy with psychological and emotional support seems to be a good idea. In earlier research, age and the husband's lack of support were significant predictors of anxiety and depression in infertile women [64]. Anxiety and depression may lead to isolation and intensify the feeling of loneliness, which is a serious issue [65]. Furthermore, the treatment strategy could be affected if depression and anxiety persist. Therefore, medical professionals should know that fewer mental and social disorders may result in better treatment outcomes. In general, women who struggle to conceive worry because they envision a future without a child to care for them in case of illness or old age. As a result, they experience social insecurity, fear of the future, fear of going through a divorce, and loneliness [66].

Similarly, this study revealed that infertile women presented with higher stress levels than infertile men in all included studies. Men may have experienced stress from fewer sources and at a lower intensity level than women, which may help to explain this discrepancy. These results align with other research on gender differences regarding stress [67]. Another possibility is that women are more frequently held responsible for infertility in marriages and experience more significant social rejection due to the pressure to conceive, which increases stress [68].

The current study demonstrated that men had a much better quality of life than women. According to earlier studies, women often report a worse adjustment to the infertile condition and higher quality of life impairments than men [69,70]. Men and women approach accepting and coping with infertility in very different ways. There is a direct correlation between having children and a woman's identity. Womanhood and motherhood go hand in hand [71], which causes quality-of-life impairments in infertile women. Moreover, infertility treatment could often be a long process that adversely impacted the quality of life of women. Some studies suggested that when the cause of infertility is the woman, the woman’s annoyance and harassment from her husband and his relatives increase, and negative feelings such as abandonment, stigmatization, and sinfulness increase in women, reducing her quality of life [39].

The study's findings demonstrated the importance of highlighting the psychological effects of infertility. Therefore, additional studies using standardized measurement techniques and with a bigger sample size are necessary to assess the emotional-psychological effects of infertility accurately.

Strengths and Limitations

An evaluation and comparison of stress, depression, anxiety, and quality of life among infertile couples are presented in this meta-analysis, taking studies from various nations into account. We used nine distinct databases for the search in the current study. The main strengths are the extensive scope of studies, and the large population examined. In addition, we found the included studies to be of good quality, yielding either high or fair-quality scores.

However, this study is not without limitations. Since this meta-analysis is based on published research, the possibility of publication bias contributed to the non-significant results being less representative. Additionally, conducting a meta-analysis on infertility is challenging due to variations in diagnosis, study methods, causes and lengths of infertility, and populations. Another drawback was using numerous measures to assess depression, anxiety, and quality of life. These various measure systems contributed significantly to the inconsistencies in this meta-analysis, making it challenging to compare the findings of other research and complicating the pooled analysis. Therefore, substantial heterogeneity, expected in meta-analysis studies, can change how results are interpreted [72]. As a result, careful consideration must be given to the present work's findings. Finally, this meta-analysis did not analyze and compare isolation, self-esteem, and suicide outcomes between infertile men and women.

## Conclusions

Infertility is a common problem, affecting one in 10 couples. It can be a challenging experience, and if left untreated, it can lead to psychological issues. To minimize psychological disruptions, it is recommended to increase the awareness of infertile people, particularly women, by offering prevention programs in counseling services and educating the public and families about new infertility treatment methods. Positive psychological states relate to treatment success, according to various research. Hence, an interdisciplinary strategy between obstetricians and psychiatrists is required to provide good-quality treatment and care for infertile patients.
